# Diagnostic Value of Echocardiography in the Diagnosis of Pulmonary Hypertension

**DOI:** 10.1371/journal.pone.0038519

**Published:** 2012-06-07

**Authors:** Christoph Hammerstingl, Robert Schueler, Lisa Bors, Diana Momcilovic, Stefan Pabst, Georg Nickenig, Dirk Skowasch

**Affiliations:** Department of Internal Medicine II, Cardiology/Pneumology, University of Bonn, Bonn, Germany; University of Cologne, Germany

## Abstract

**Aims:**

To determine the value of echocardiography including tissue Doppler imaging (TDI) and right ventricular (RV) speckle tracking analysis for the diagnosis of pulmonary hypertension (PH) and discrimination between pre- and postcapillary PH.

**Methods and Results:**

155 consecutive patients (mean age 70.5±13.0 years, 81 [52%] male gender, BMI 27.2±6.1 kg/m^2^) with PH undergoing right heart catheterization (RHC) and transthoracic echocardiography (TTE) with TDI between January 2008 and December 2009 were retrospectively evaluated including offline speckle tracking analysis of RV contractility. After RHC 23.2% of patients (36) were diagnosed with precapillary PH. Invasive results from RHC were significantly correlated to TTE measurements (E/é, postcapillary wedge pressure [PCWP], r = 0.61, P<0.001; mean, systolic pulmonary arterial pressure [mPAP, sPAP], r = 0.43, P<0.001). Single echocardiographic parameters were of good predictive value for final PH-diagnosis (sPAP, area under the curve [AUC] 0.63, P = 0.025; lateral apical RV longitudinal strain [RVaSl)], AUC 0.76, P = 0.001; E/é, AUC 0.84, P<0.001) which could be increased by combining most predictive parameters after receiver operating curves (ROC) cut off analysis (sPAP>69 mmHg, E/é<12, RVaSl ≥−8.4%). TTE had a sensitivity of 33.33% and a specificity of 100% to identify patients with precapillary PH, and a negative predictive value of 84.72% to rule out precapilary PH.

**Conclusion:**

Echocardiography allows feasible and reliable estimation of PH and seems helpful to distinguish between pre-and postcapillary PH. Further prospective studies on patients with different manifestations of PH must validate the predictive value of echocardiography in this clinical setting.

## Introduction

Precapillary pulmonary arterial hypertension (PAH) is a severe and life threatening disease, leading to death if untreated within 2–3 years after diagnosis [1]. Definite diagnosis of PAH is often delayed with a median of 3 years from onset of first symptoms to start of effective therapy [2]. Therefore, current guidelines emphasize the importance of early diagnosis of precapillary PAH and differentiation from other entities of pulmonary hypertension (PH) [3]. Up to now definite diagnosis of PAH can only be confirmed by invasive right heart catheterization (RHC) which is not widely available, puts the patients at risk for rare but serious complications, and which is inconvenient for frequent follow up evaluations [4]. Furthermore, PAH is a rare disease and invasive RHC confirms postcapillary PH in most cases with elevated pulmonary pressure. TTE is a non-invasive method which is widely available, cost effective, and has been established as non-invasive screening technique for the detection of PH. Furthermore, echocardiographic studies are recommended at regular intervals during the follow up of patients with proven PAH under specific therapy [5]. It would be of great interest to identify a simple, non-invasive diagnostic algorithm which allows for (a) adequate PH diagnosis with a sufficient high reliability, and (b) discriminates between pre-and postcapillary PH.

Therefore, we sought to determine the value of an echocardiographic algorithm for the diagnosis of PH in patients with typical clinical symptoms which were transferred to our hospital for further evaluation.

## Results

155 consecutive patients with PH undergoing standardized two dimensional TTE before RHC between January 2008 and December 2009 were analysed. Baseline characteristics as well as results from RHC and TTE are presented in ***table 1***. After RHC 36 (23.2%) patients were diagnosed with precapillary PH; in 119 (76.8%) patients PH was found to be postcapillary.

**Table 1:** Baseline data, groups divided with regard to findings from right heart catheterization.

	All patients(n = 155)	PrecapillaryPH (n = 36)	PostcapillaryPH (n = 119)	*p-value*
Age (years)	70.5±13.0	65.9±16.8	71.3±11.4	**0.002**
BMI (kg/m^2^)	27.2±6.1	26.2±6.0	27.6±6.4	0.37
Male gender (n, %)	81 (52%)	13 (36%)	68 (57%)	**0.03**
Hypertension (n, %)	91 (59%)	12 (36%)	78 (65%)	**0.002**
Diabetes mellitus (n, %)	33 (21%)	7 (19%)	26 (22%)	0.82
Smokers (n, %)	29 (19%)	7 (19%)	22 (18%)	1.0
Stroke (n, %)	3 (2%)	0 (0%)	3 (2%)	1.0
Lipid disorder (n, %)	45 (29%)	5 (14%)	40 (33%)	**0.02**
CAD (n, %)	80 (51%)	14 (39%)	66 (55%)	0.09
NYHA FC II (n, %)	28 (18%)	4 (11%)	24 (20%)	0.25
NYHA FC III (n, %)	93 (60%)	27 (75%)	66 (55%)	0.47
NYHA FC IV (n, %)	34 (22%)	5 (14%)	29 (24%)	0.31
*Medication*				
Aspirin (n, %)	89 (57%)	17 (47%)	72 (60%)	0.18
ß-blocker (n, %)	115 (74%)	16 (44%)	99 (83%)	**<0.0001**
ARB/ACEI (n, %)	24 (15%)	3 (8%)	21 (18%)	0.29
Diuretics (n, %)	128 (83%)	23 (64%)	105 (88%)	**0.002**
Statin (n, %)	86 (55%)	14 (39%)	72 (60%)	**0.03**
CCB (n, %)	30 (19%)	8 (21%)	23 (19%)	1.0
OAC (n, %)	64 (41%)	12 (33%)	52 (44%)	0.33
*Right heart catheterization*				
mPAP [mmHg]	38.7±10.7	39.3±13.3	38.5±9.8	0.70
sPAP [mmHg]	55.0±17.6	60.3±17.4	53.5±17.4	**0.04**
CO [l/min]	3.6±3.8	3.2±2.3	3.4±4.2	0.42
PCWP [mmHg]	22.9±9.3	11.2±2.6	26.6±7.3	**<0.0001**
RV systolic pressure [mmHg]	54.2±20.1	58.1±18.8	53.1±20.5	0.18
RV diastolic pressure [mmHg]	5.3±5.8	4.3±5.8	5.6±5.8	0.24
RV mean pressure [mmHg]	9.4±8.6	11.9±12.1	8.6±7.1	**0.04**
RA mean pressure [mmHg]	13.5±13.1	12.7±5.4	13.8±14.7	0.69
*Echocardiography*				
sPAP [mmHg]	51.8±17.1	58.3±23.6	49.9±14.2	**0.009**
LVEF [%]	53.2±18.1	61.8±13.9	50.6±18.5	**0.001**
LAV [ml]	84.0±52.7	79.9±64.9	85.2±48.7	0.6
RVDs [cm]	2.4±1.1	2.4±1.2	2.4±1.1	0.8
RVDd [cm]	3.3±1.4	3.4±1.6	3.3±1.3	0.88
E/é	16.5±7.9	9.9±2.9	18.5±7.9	**<0.0001**
MV E [m/s]	1.1±0.7	0.9±0.5	1.2±0.8	0.18
MV A [m/s]	0.6±0.5	0.6±0.5	0.6±0.5	0.77
MV é [m/s]	0.07±0.06	0.07±0.04	0.07±0.07	0.79
MV Á [m/s]	0.07±0.04	0.07±0.04	0.07±0.05	0.80
é/Á	1.3±0.8	1.5±0.9	1.2±0.7	0.07
E/A	1.5±1.2	1.1±0.9	1.7±1.2	**0.01**
IVSd [cm]	1.2±0.6	1.1±0.3	1.3±0.6	0.08
2D RV longitudinal lateral apical strain [%]	−8.0±4.6	5.7±3.4	−10.4±4.5	**0.003**

BMI, body mass index; CAD, coronary artery disease; NYHA FC, NYHA functional class; CCB, calcium channel blocker; ARB/ACEI, angiotensin receptor blockers/angiotensin converting enzyme inhibitor; mPAP, mean pulmonary arterial pressure; sPAP, systolic pulmonary arterial pressure; PCWP, pulmonary capillary wedge pressure; RV, right ventricle; RA, right atrium; LVEF, left ventricular ejection fraction; E, early; A, atrial; MV, mitral velocity; RVDs/d, systolic/diastolic right ventricular diameter; LAV, left atrial volume; IVSd, diastolic interventricular septum thickness.

Echocardiographically determined sPAP was >30 mmHg in all patients leading to a sensitivity of this parameter of 100% for the identification of patients with PH; however, sPAP alone can not discriminate pre-from postcapillary PH; its specificity was 0%.

**Figure 1:**
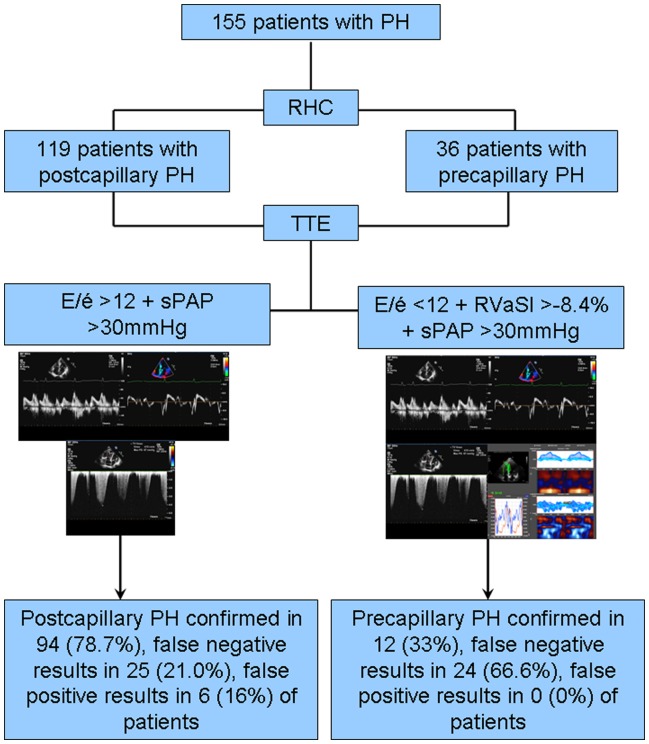
Incremental diagnostic value of apical strain and E/é in addition to echocardiographically determined sPAP for identification of patients with precapillary PH. *AUC, area under the curve; CI, confidence interval; sPAP, systolic pulmonary arterial pressure; RVSl, right ventricular longitudinal strain.*

When compared with results from RHC, non-invasive determined sPAP was significantly correlated with invasively measured mPAP (r = 0.43; P<0.0001), but not with invasive sPAP (r = 0.15, P = 0.06). PCWP values from RHC were significantly correlated with TTE derived E/é values (r = 0.61, P<0.0001).

When comparing patient groups based on final PH diagnosis several echocardiographic parameters and demographic parameters showed significant differences (***table 1***). Patients with precapillary PH tended to have a lower LV EF, lower E/é values, higher sPAP and higher RVaSl values. Mean values of invasively measured sPAP (60.3±17.4 mmHg vs. 53.5±17.4 mmHg, P = 0.04) and sPAP derived from echocardiography (58.3±23.6 mmHg vs. 49.9±14.2 mmHg, P = 0.009) were significantly different in patients with precapillary and postcapillary PH. Interestingly, only in patients with precapillary PH, there was a significant correlation between invasive and non-invasive measurements of sPAP-values (TTE: 58.3±23.6 mmHg, RHC: 60.3±17.4, mmHg; P = 0.008; r = 0.37), whereas in patients with postcapillary PH this correlation was not found to be significant (TTE: 49.9±14.2 mmHg, RHC: 53.5±17.4 mmHg; P = 0.07; r = 0.15).

### Echocardiographic Parameters Identifying Subjects with Postcapillary PH

When comparing groups with regard to invasive findings from RHC only in patients with postcapillary PH a significant correlation was found between PCWP and E/é (r = 0.44, P<0.001 vs. r = 0.07, P = 0.67), with increasing E/é values in patients with postcapillary PH. ROC curve analysis confirmed the predictive value of this parameter to identify patients with postcapillary PH (AUC, 0.84, P<0.001) with an optimal cut off value of >12. This parameter alone had a sensitivity of 78.69% and specificity of 81.82%, a positive predictive value of 94.12%, and a negative predictive value of 50.94% to identify patients with postcapillary PH.

### Echocardiographic Parameters Identifying Subjects with Precapillary PH

Logistic stepwise backwards regression analysis of most predictive variables after univariate regression analysis (LV EF, E/A, sPAP, E/é and RVaSl) identified only E/é (P = 0.003) and RVaSl (P = 0.004) to be independently associated with the presence of precapillary PH.

This finding was confirmed by ROC analsis of RVaSl, (AUC 0.76, P = 0.001; cut off value, >−8.4%) with a sensitivity of 33.33% and a specificity of 95.08% of this parameter for the identification of patients with precapillary PH.

When combining sPAP with RVaSl, TTE showed a sensitivity of 88.24%, and specificity of 68.75% to identify patients with precapillary PH. Finally, when adding sPAP to RVaSl and E/é the diagnostic value of echocardiography for the diagnosis of precapillary PH showed a sensitivity of 33.33% and a specificity of 100%, a positive predictive value of 100% and a negative predictive value of 84.72% (***figure 1***).

## Discussion

In our study we aimed to evaluate the diagnostic value of echocardiography as non invasive screening method in patients with suspected PH by using a simple algorithm which was evaluated in a large cohort of symptomatic patients undergoing non-invasive and invasive testing. The most important findings of our study are that (a) estimation of PCWP with echocardiography is feasible and of high value to identify subjects with postcapillary PH and (b) echocardiography in combination with 2D speckle tracking analysis of RV deformation capability has a high predictive value to identify patients with precapillary PH.

### Echocardiographic Parameters Predicting Postcapillary PH

Hadano could show a relevant correlation of E/é ratio with PCWP and LVEDP, respectively in patients with heart failure undergoing RHC which correlates with the results from our study. However, this group did not investigate the diagnostic value of this parameter in patients with suspected PH but without known structural heart disease. [6], [7].

More recently, Ruan and colleagues found normal or reduced E/é ratios in 70 patients with primary PH and increasing E/é values in patients with PH due to cardiac aetiology were similarly to the findings in our study group [8].

In contrast to this study group who suggested the use of lateral mitral annulus systolic velocities [8] we used the average of septal and lateral mitral annulus velocities as recommended by the American and European society of echocardiography [9], [10]. Additionally, we were able to show the high discriminative value of E/é to distinguish pre-from postcapillary PH in a considerably larger patient group.

### Echocardiographic Assessment of Patients with Precapillary PH

Transthoracic echocardiography provides several different variables which correlate well with right heart hemodynamics and function, and therefore should always be performed in the case of suspected PH [2]. However, up to now echocardiography alone is considered inaccurate for the determination of pulmonary arterial pressure in symptomatic subjects due to its limited sensitivity and specificity [11] and, moreover, TTE is supposed unable to differentiate between pre-and postcapillary PH, as suggested in a recently published study by Bonderman et al. [12]. This group evaluated a non-invasive algorithm to exclude precapillary pulmonary hypertension (PH) in 372 (251 retrospective, 121 prospective subjects) symptomatic patients with PH combining echocardiography, ECG and laboratory testing (NT-proBNP). In this study on highly selected patients the non-invasive diagnostic algorithm including TTE had a good sensitivity of 100% and a low specificity of 19.3% for the detection of precapillary PH [12].

In our study TTE derived sPAP did not correlate well with sPAP from RHC. This finding may partly be explained by to the fact that all patients underwent TTE ≥1 day before invasive measurements and were fasting for at least 12 hours before undergoing RHC leading to volume depletion and a decrease in sPAP values. Importantly, invasive and non-invasive sPAP were not significantly different in patients with precapillary PH (60.3±17.4 mmHg, 58.3±23.6 mmHg, P = 0.64), but in patient with postcapillary PH after RHC (53.5±17.4 mmHg, 49.8±14.2 mmHg, P = 0.04), indicating on a stronger volume-load dependency of PAP in patients with postcapillary PH. These finding supports current PH guidelines [2] suggesting a systemic volume challenge in patients with suspected postcapillary PH when invasive results are not decisive.

**Figure 2:**
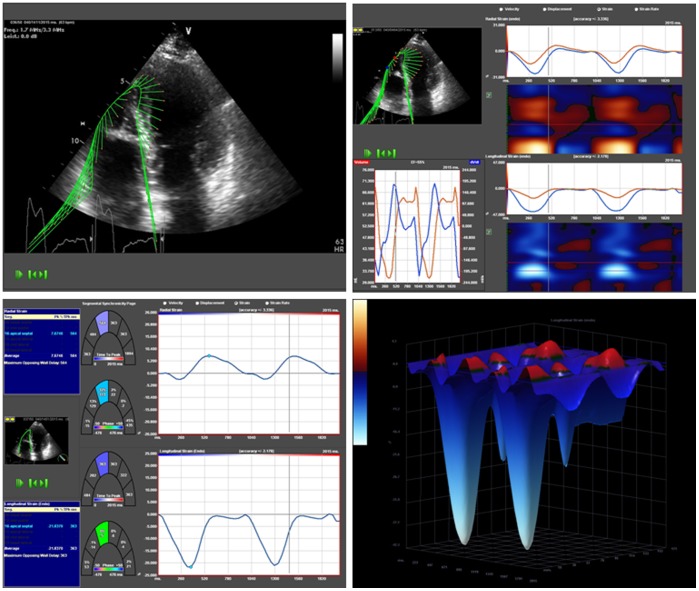
Patient distribution according to results of right heart catheterization; flow chart of the study design. *PH, postcapillary pulmonary hypertension; RHC, right heart catheterization; TTE, transthoracic echocardiography; sPAP, systolic pulmonary arterial pressure; RVaSl, apical right ventricular longitudinal strain.*

### RV Strain Analysis in Patients with Pulmonary Hypertension

Studies on 2D RV speckle tracking in patients with PH are scarce. Ahmad et al. showed in 63 patients with different aetiologies of RV impairment that 2D ST derived RV strain correlated well with global RV function as determined with cardiac magnetic resonance imaging (cMRI) [13].

In another study Fukuda and colleagues examined 45 patients with precapillary PH and found RV free wall longitudinal strain values to be of high diagnostic value of for the determination of RV function before and after pharmacological treatment. Interestingly, in this study RV free wall longitudinal strain values was highly significant correlated with changes in 6 minute walking distance [14].

This is the first study to evaluate the value the combination of non-invasive measurements for the estimation of PCWP, PAP, and RVaSl to diagnose precapillary PH in symptomatic patients with suspected PH. We found echocardiography to be of good sensitivity and specificity for the detection of precapillary PH and to be of reasonable good value to rule out precapillary PH which is of great importance in this patient cohort.

### Limitations

Our study cohort consisted of patients with invasively proven PH. We did not follow a control group and our study design was retrospective. However, our results indicate on a high reliability of echocardiographic diagnostic in the setting of PH and suggest a high reliability of this non invasive strategy for the differentiation of pre-and postcapillary PH.

We did not evaluate the underlying aetiology of PAH or postcapillary PH in this study. Sensitivity and specificity of this test might not be the same in different patient groups with rare aetiologies of PH. Therefore, further prospective studies on different patients populations are required to confirm our findings.

### Clinical Impact

RHC is often delayed in symptomatic patients with suspected PH. Echocardiography provides reliable estimation of pulmonary pressure and left ventricular filling pressures and is therefore a valuable tool for the assessment of PH in clinical symptomatic patients. Since the combination of sPAP, RVaSl and E/é was of high specificity to identify patients with precapillary PH, this non invasive approach including RV speckle tracking analysis might be an appropriate tool during the diagnostic work up of patients with suspected PH.

### Conclusion

Echocardiography allows feasible and reliable estimation of PH and seems helpful to distinguish between pre-and postcapillary PH. Further prospective studies on patients with different manifestations of PH must validate the predictive value of echocardiography in this clinical setting.

## Methods

### Ethics Statement

The study has been approved by the ethics committee of the University of Bonn. Informed consent was obtained by all patients before study inclusion. Furthermore all the data were analyzed anonymously. This study has been conducted according to the principles expressed in the Declaration of Helsinki.

### Study Concept

***Figure 2*** shows a flow chart of our study concept. Consecutive patients with suspected PH undergoing standardized two dimensional TTE ≤7 days before RHC were retrospectively analysed. Based on the invasive hemodynamic evaluation during RHC, precapillary or postcapillary PH was diagnosed according to current guidelines [3]. Patients with significant valvular heart disease and cardiomyopathies of any aetiology were excluded from the analysis. Precapillary PH was defined as mean pulmonary arterial pressure (mPAP) ≥25 mmHg and pulmonary capillary wedge pressure (PCWP) of ≤15 mmHg; patients with an Mpap ≥25 mmHg and a PCWP >15 mmHg were defined as suffering from postcapillary PH.

The following echocardiographic parameters were used to evaluate the presence of PH and the concomitant finding of an elevated PCWP which mainly distinguishes postcapillary PH from precapillary PH.

*Systolic pulmonary artery pressure* (sPAP) was estimated with continuous wave Doppler of systolic tricuspid regurgitant velocity and considered elevated when reaching values >30 mmHg. *PCWP* was assumed to be elevated (>15 mmHg) in cases with E/é ratio >12, a echocardiographic parameter which reflects on left ventricular filling pressure and is defined as the ration of peak mitral inflow velocities and peak mitral annulus velocity. Subjects with sPAP>30 mmHg and E/é <12 were assumed to have precapillary PAH, whereas in patients with sPAP>30 mmHg and E/é >12 PH was considered postcapillary. Furthermore, offline speckle tracking (ST) analysis of right ventricular (RV) contractility was performed in all subjects and compared with the results from RHC aiming to determine the diagnostic value of this new imaging technique in this clinical setting.

**Figure 3:**
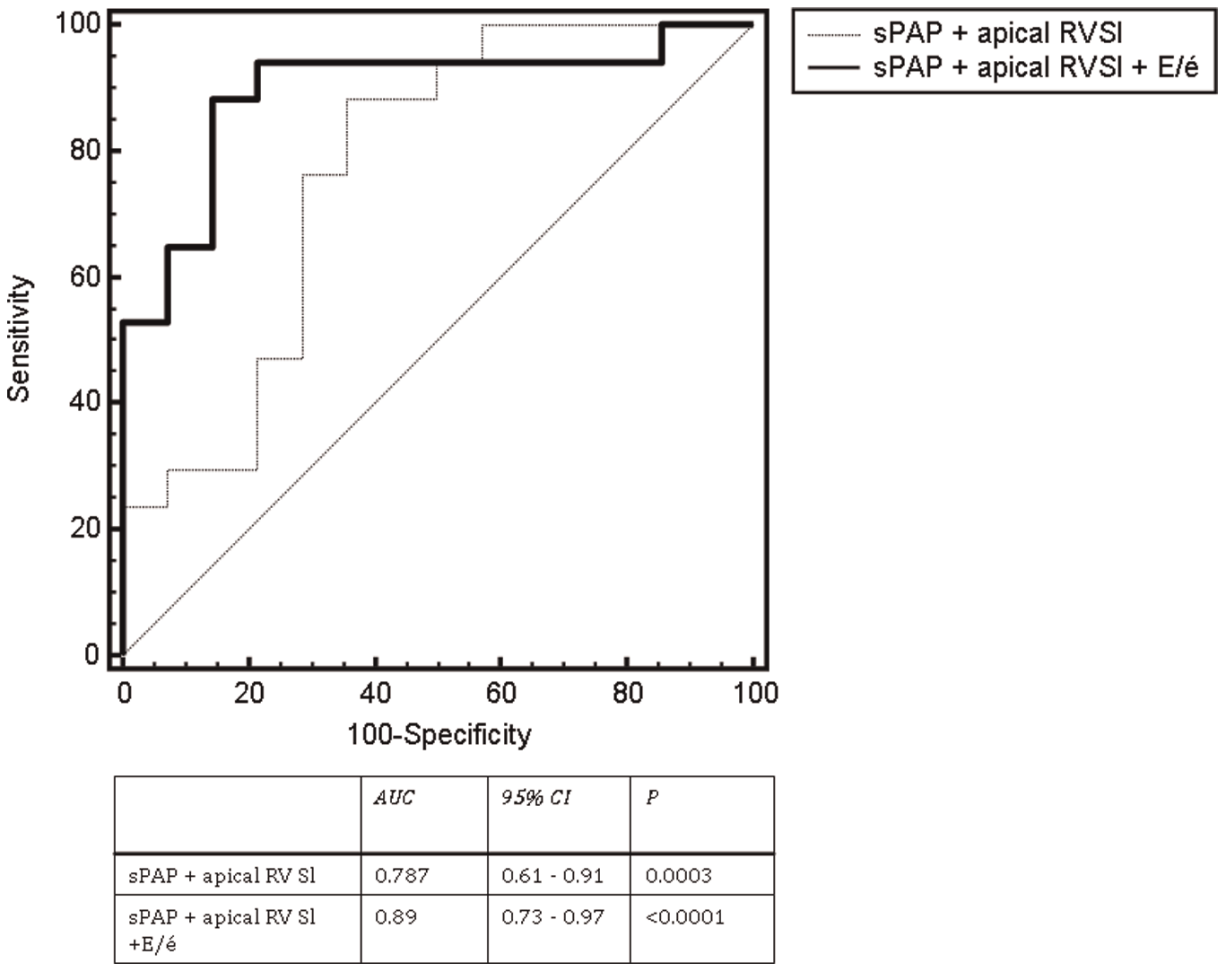
Right ventricular speckle tracking and 3 dimensional visualisation of longitudinal right ventricular strain values. *Right ventricular; 3D, three dimensional; RVSl, right ventricular longitudinal strain.*

### Transthoracic Echocardiography

Each patient underwent standardized two-dimensional transthoracic echocardiography for the determination of left and right ventricular functional parameters and dimensions according to the recommendations of the American Society of Echocardiography [15], [16] using commercially available ultrasound scanner (Vivid 7, General Electric Medical Health, Waukesha, Wisconsin, USA; iE 33, Philips Medical Systems, Koninklijke N.V.) with a 2.5-MHz phased-array transducer. Echocardiographic views, including apical four-and two-chamber views (4CV,2CV) and parasternal (long-and short-axis views), with the patient in the left lateral decubitus position, were obtained in two-dimensional, three-dimensional and colour tissue Doppler imaging (TDI) modes. 2D ejection fraction (EF) was calculated by Simpsońs rule from 4 CV. Mitral inflow velocities were recorded by standard pulsed-wave Doppler at the tips of the mitral valve leaflets in an apical 4CV. E/é ratio was determined by measuring TDI derived systolic and diastolic velocities of the septal and lateral mitral valve annulus and averaging the values and conventional measurement of mitral valve inflow velocities according to recommendations of the American Society of Echocardiography [9]. During echo sPAP was estimated by measuring the peak systolic tricuspid regurgitant velocity flow at continuous wave Doppler if applicable.

### 2D Speckle Tracking Analysis of Right Ventricular Deformation Capabilities

***Figure 3*** shows an example of RV ST analysis of RV deformation capabilities. Two cine loops from apical four chamber view were digitized and stored on an echocardiographic imaging server (XCELERA, Philips Medical Systems, Koninklijke N.V.). Offline 2D ST analyses of the gray scale images obtained by 2D echocardiography were done by using commercially available software (TomTec Imaging Systems GmbH, Unterschleissheim, Germany). The endocardium of the free RV wall was manually traced starting from the lateral tricuspidal annulus to RV apex, and was tracked by the 2D strain software along the border throughout two cardiac cycles. Accuracy of border tracking was manually verified and adjusted if needed. The free right ventricular wall was segmented visually in a basal, midventricular and apical segment. For the determination of regional RV impairment we followed the approach of Dambrauskaite [17] and Lopez-Candalez [18]. Therefore only longitudinal lateral apical RV Sl (RVaSl) entered further analysis. Of note, RV speckle tracking analysis of free RV wall was applicable in all patients.

### Statistical Analysis

Exploratory data analysis was performed and no adjustment was made for multiple tests.

Normal distribution of continuous variables was examined using the Kolmogorov – Smirnov test. Continuous data was expressed as mean values±standard deviation. Two-tailed P-values were calculated and considered to be significant if ranging below 0.05. Comparisons between two groups were performed with using students t test for paired samples or pair wise comparisons with Wilcoxon signed rank test for paired continuous variables. For categorical data Fisher’s exact test were calculated. Statistics were performed using SPSS for Windows (PASW statistic., Version 17.0.2, SPSS Inc., Chicago, Illinois, USA) and MedCalc statistical software (MedCalc Software, Version 11.4.1.0, Mariakerke, Belgium).

To assess the predictive ability of the echocardiographic parameters logistic regression was performed. Associated receiver operating characteristic (ROC) curves for predicted probabilities were drawn for a diagnostic model including sPAP, 2D RVaSl and E/é. The corresponding areas under the curve along with their 95% CIs were calculated. Furthermore, we aimed to determine the diagnostic value of single parameters, as well as the combination of most predictive parameters for PH diagnosis by calculating the sensitivity, specificity, negative and positive predictive values for each parameter and its combination.
